# Selective Endothelial Overexpression of Arginase II Induces Endothelial Dysfunction and Hypertension and Enhances Atherosclerosis in Mice

**DOI:** 10.1371/journal.pone.0039487

**Published:** 2012-07-19

**Authors:** Boris L. Vaisman, Karen L. Andrews, Sacha M. L. Khong, Katherine C. Wood, Xiao L. Moore, Yi Fu, Diane M. Kepka-Lenhart, Sidney M. Morris, Alan T. Remaley, Jaye P. F. Chin-Dusting

**Affiliations:** 1 Cardiovascular-Pulmonary Branch, National Heart, Lung, and Blood Institute, National Institutes of Health, Bethesda, Maryland, United States of America; 2 Vascular Pharmacology Laboratory, Baker IDI Heart and Diabetes Institute, Melbourne, Victoria, Australia; 3 Departments of Microbiology and Molecular Genetics, University of Pittsburgh, School of Medicine, Pittsburgh, Pennsylvania, United States of America; Idaho State University, United States of America

## Abstract

**Background:**

Cardiovascular disorders associated with endothelial dysfunction, such as atherosclerosis, have decreased nitric oxide (NO) bioavailability. Arginase in the vasculature can compete with eNOS for L-arginine and has been implicated in atherosclerosis. The aim of this study was to evaluate the effect of endothelial-specific elevation of arginase II expression on endothelial function and the development of atherosclerosis.

**Methodology/Principal Findings:**

Transgenic mice on a C57BL/6 background with endothelial-specific overexpression of human arginase II (hArgII) gene under the control of the Tie2 promoter were produced. The hArgII mice had elevated tissue arginase activity except in liver and in resident peritoneal macrophages, confirming endothelial specificity of the transgene. Using small-vessel myography, aorta from these mice exhibited endothelial dysfunction when compared to their non-transgenic littermate controls. The blood pressure of the hArgII mice was 17% higher than their littermate controls and, when crossed with apoE −/− mice, hArgII mice had increased aortic atherosclerotic lesions.

**Conclusion:**

We conclude that overexpression of arginase II in the endothelium is detrimental to the cardiovascular system.

## Introduction

Arginase, a critical hepatic enzyme in the urea cycle, catalyses the conversion of L-arginine to urea and ornithine. It exists in two isoforms: arginase I in the cytoplasm and arginase II in the mitochondria. In general, vascular arginase is thought to compete with endothelial nitric oxide synthase (eNOS) for L-arginine. As such, upregulation of arginase activity and expression has been reported to play a role in various vascular pathologies, such as pulmonary hypertension associated with sickle cell disease [1], primary pulmonary arterial hypertension, [2] ischemia-reperfusion, [3] uremia, [4] as well as various animal models of arterial hypertension, [5] aging, [6] sexual arousal, [7], [8] diabetes [9] and atherosclerosis [10], [11], [12].

The first indication that arginase may play a role in atherogenesis came from reports that apoE −/− mice fed a high fat diet have increased aortic arginase activity and that inhibition of arginase resulted in reduced plaque size [11]. In addition, the proatherogenic oxidized low density lipoproteins have been reported to stimulate arginase II activity and attenuate NO production in human endothelial cells [12]. More recently, studies examining the effect of high fat and high cholesterol diets on systemic L-arginine bioavailability and arginase activity suggest that arginase may in fact contribute to the initiation of atherosclerosis [13]. Conversely, increased levels of arginase I in macrophages have been linked with atheroprotection [14] and regression of atherosclerotic plaques [15].

To date, a lack of selective pharmacological inhibitors has hindered investigations into the role of the specific arginase isoforms in blood vessel pathophysiology. Although several arginase inhibitors exist, including *N^G^*-hydroxy-L-arginine (L-NOHA), N^ω^-hydroxy-nor-arginine (nor-NOHA), L-valine, nor-valine, α-difluoromethylornithine (α-DFMO), (S)-(2-boronoethyl)-L-cysteine-HCl (BEC) and 2(s)-amino-6-boronohexanoic acid (ABH) [16], [17], none of these distinguish between arginases I and II. Furthermore, the use of many of these compounds is severely limited in functional vascular studies due to their vasodilator properties [18], such that blockade of more than just arginase is apparent. Given these limitations, genetic manipulation techniques offer an alternative way to examine the contributory roles of arginase I and II in vascular function. Mice globally lacking arginase I do not survive beyond 10–14 days post-birth due to the systemic build-up of toxic ammonia [19]. Arginase II-deficient mice, on the other hand, have lifespan identical to their WT controls but were found unexpectedly to have hypertension, a phenotype that misaligns with their dampened local vasoconstrictory profile and thus limits the usefulness of this model in cardiovascular studies [20]. To overcome these limitations, transgenic mice with endothelial cell specific overexpression of arginase II were generated. Here, we report on the role of arginase II on endothelial function, blood pressure and in the pathogenesis of atherosclerosis.

## Methods

### Creation of hArgII Mice and Crossbreeding with apoE −/−

The study protocol was approved by the by the Animal Care and Use Committee of the NHLBI (#H-0050R1) and by Alfred Medical Research and Education Precinct (AMREP) Animal Ethics Committee (#E/0710/2008/B), which adheres to the National Health and Medical Research Council (NHMRC) of Australia Code of Practice for the Care and Use of Animals for Scientific Purposes.

The full-length hArgII (2 kb) cDNA flanked by Not I linkers was inserted into the unique Not I cloning site of the endothelial specific expression vector pSPTg.T2FpAXK [21], [22] (kindly provided by Dr. Thomas N. Sato; The University of Texas, Southwestern Medical Center at Dallas, TX). The vector has 2.1 kb of the mouse Tie2 promoter, SV40 polyA signal and a 1.6 kb Tie2 enhancer. Correctly oriented plasmids were digested with Sal I, and DNA fragments containing the Tie2 promoter, hArgII cDNA, the SV40 polyA signal and Tie2 enhancer were isolated by preparative electrophoresis in 0.8% agarose gel and Zymoclean gel DNA recovery kit. The DNA fragments were additionally purified by ultracentrifugation in a CsCl gradient in a Beckman TL-100 table top ultracentrifuge at 95000 RPM for 24 hrs at 20°C and dialyzed against 10 mM Tris-HCl, pH 7.4, 0.1 mM EDTA. Microinjections were performed into the pronuclei of fertilized eggs from C57BL/6J females (Jackson Laboratory, ME, USA). The transgenes were transferred on to the apoE−/− background by crossing carriers of the hArgII with apoE−/− mice on the C57Bl/6J background (Jackson Laboratory, Stock Number 002052).

All mice were fed *ad libitum* a standard rodent autoclaved chow diet containing 4% fat and 50 mg cholesterol (NIH31 normal chow diet; Zeigler Brothers Inc., Gardners, Pennsylvania, USA). Both male and female transgenic mice (aged 3–8 months) and their WT littermate controls were used in this study.

### PCR Genotyping of Mouse Colonies

For genotyping, DNA was isolated from tail clips and analyzed by real-time quantitative PCR (qPCR) on an ABI7300 or ABI7900HT, using primers specific for hArgII or m18sRNA (ABI TaqMan MGB assays Hs00968978_m1 and Hs00982837_m1). DNA isolated for injections was used to make calibration curves. The following standard conditions were used during qPCR: volume of reagent mix, 20 µl for ABI7900HT and 25 µl for ABI7300, concentration of DNA in wells was 0.2 ng/ul. ABI TaqMan Universal PCR Master Mix, No AmpErase UNG, Cat# 4324018 was used. The final concentration of primers was 900 nM, TaqMan MGB probe −250 nM. The manufacturer’s thermal cycler protocol (95 C for 10 min, followed by 40 cycles at 95°C for 15 s, 60°C for 1 min) was used.

### Western Blot Analysis

Kidney, heart and aortic lysates were obtained by homogenization in a glass homogenizer in cold lysis buffer then centrifuged at 13000 × g for 10 mins to removed unlysed cell bodies. Western blot analysis was done as described previously, [23] using 6, 8 or 12% acrylamide gels for eNOS monomer:dimer, arginase II and eNOS/iNOS, respectively. Twenty (eNOS monomer:dimer), fifty (eNOS/iNOS) or one hundred (arginase) µg of protein were loaded per lane. Rabbit polyclonal arginase II antibody cross reactive to mouse (Santa Cruz; 1∶3000), mouse monoclonal eNOS or iNOS antibody (BD Biosciences; 1∶2500) were utilized. A goat anti-rabbit (for arginase II) and a goat anti-mouse (for eNOS/iNOS) IgG-HRP secondary antibody (Bio-Rad, 1∶3000 dilution, one hour incubation) were used for detection. The eNOS monomer:dimer ratio was determined using low-temperature SDS-PAGE (LT-PAGE) where gels and buffers were kept at 4°C prior to and during electrophoresis. Following LT-PAGE, gels were transferred on to a PVDF membrane at 4°C and the blots were probed as routine western blots for eNOS monomer and dimer (both BD Transduction; 1∶10,000) [24]. To ensure equal loading, blots were probed with β-tubulin antibody (Santa Cruz 1∶1000). Bands of interest were visualized using SuperSignal West Pico reagent (Pierce, Rockford, IL). Immunoreactive bands were quantified by densitometry using image J (version 1.42q).

### Expression Analysis with Real-time Quantitative RT-PCR

**Figure 1:**
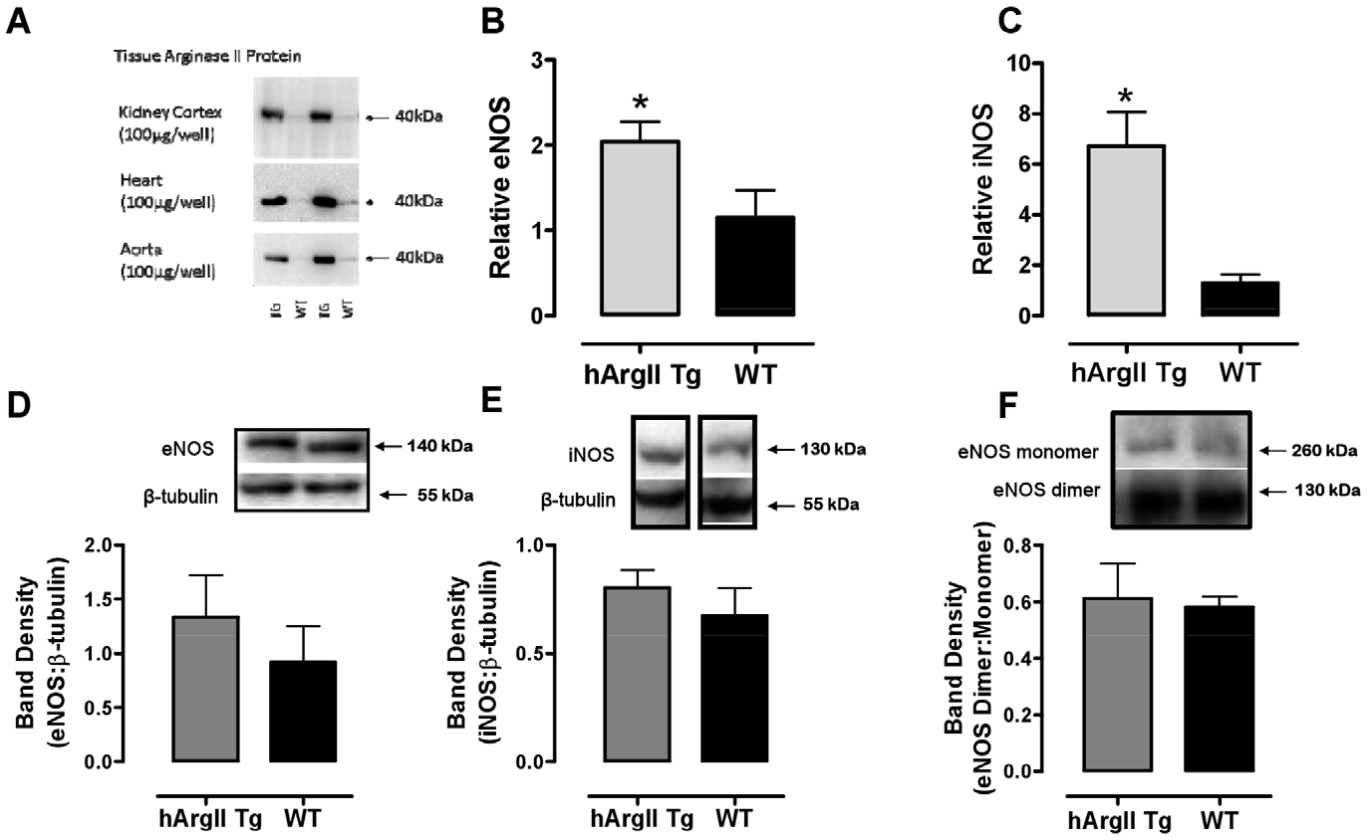
Representative western blot analysis of arginase II in A kidney, heart and aorta of male homozygous Line 11 hArgII mice (TG) and their WT littermate controls. Expression analysis of **B** eNOS and **C** iNOS using real-time quantitative RT-PCR and protein expression of **D** eNOS and **E** iNOS and **F** eNOS monomer:dimer ratio in aorta from male homozygous Line 11 hArgII mice and their WT littermate controls. Data are presented as mean ± SEM; n = 4−8. Values were compared with an unpaired Student’s t-test where * = P<0.05.

Tissue samples were preserved in RNAlater (Invitrogen, Cat. No. AM7020) until RNA isolation, using TRIzol Reagent (Invitrogen, Cat. No. 15596-026). Isolated RNA (2 µg) was treated with DNAse (Promega) and then reverse transcribed into cDNA using ABI TaqMan Reverse Transcriptase Reagents Kit (Cat. No. N808-0234). 40 or 50 ng cDNA was used for per qPCR reaction. PCR reaction, either TaqMan (Table 1) or SYBR Green assay (Fig. 1), was respectively performedon the ABI7300 or ABI7900HT. Primers and probes for Taqman reaction were obtained either from ABI kits or designed by using ABI Primer Express 3.0 software, while primers for SYBR Green were designed using Roche’s ProbeFinder software and synthesized by Sigma. All sequences are available upon request. Two methods to calculate expression levels were used. For Taqman assay he relative expressions of hArgII, as well as of other genes, were calculated against the expression of mouse arginase II gene in the kidney of non-transgenic mice. Expression of mouse iNOS and eNOS, measured by SYBR Green assay, were calculated by comparative C_T_ (ΔΔC_T_) method with 18sRNA used for normalization.

**Table 1:** Expression of hArgII, mArgI and mArgII mRNAs in tissues of female C57Bl/6NT and hArgII transgenic mice.

Mice	Gene	Relative expression (%)				
		Aorta	Heart	Kidney	Liver	Lung
**hArgII** **transgenic**	hArgII	859±163	1741±450	550±160	106±24	1086±247
**WT**	mArgII	0.2±0.05	0.3±0.03	100±7.9	1.2±0.6	11.2±2.5
**hArgII Tg**		0.1±0.01	0.1±0.05	150.3±50.7	2.5±1.4	12.1±0.5
**Difference** **(p)**		NS	NS	NS	NS	NS
**WT**	mArgI	3.6±3.3	7.9±5.5	1.2±0.9	5320±254	11.2±3.1
**hArgII Tg**		2.7±1.2	1.6±0.6	1.4±0.6	6224±597	30.2±18.6
**Difference** **(p)**		NS	NS	NS	NS	NS

Expression of hArgII, mArgI and mArgII in five tissues (aorta, heart, kidney, liver and lung) of heterozygous Line 4 hArgII mice (n = 3) and C57Bl/6NTac (n = 3). All data were presented as percent relative to expression of mArgII in normal kidney, which was assigned as 100%. Values are given as mean ± SEM and compared using Student’s t-test where NS represents not significant.

### Analyses of Plasma Lipid, Lipoproteins and L-arginine Metabolites

Total cholesterol, triglycerides, phospholipids, and free cholesterol in EDTA-plasma were quantified after a 4-hour fast, using enzymatic kits as previously described [25]. HDL cholesterol (HDL-C) was determined as the cholesterol remaining in plasma after precipitation of apoB-containing lipoproteins with Raichem HDL-Cholesterol reagent (cat. No. 82051). Plasma concentrations of L-arginine and its metabolites, citrulline, *N^G^*-hydroxy-L-arginine (NOHA), asymmetric dimethylarginine (ADMA), symmetric dimethylarginine (SMDA), and ornithine were determined by high-performance liquid chromatography (HPLC), as previously described [26].

### Mouse Peritoneal Macrophages

Resident peritoneal macrophages were obtained by peritoneal lavage [27] with 10 ml of sterile PBS without Ca^2+^ and Mg^2+^ containing 50 µg/ml of heparin. Cells from 10 mice were placed into 5 wells of 6-well tissue culture plates and allowed to adhere for 1.5 hr in serum-free DMEM or RPMI-1640, after which non-adherent cells were removed by rinsing the cells with PBS without Ca^2+^ and Mg^2+^. Adherent cells were directly used for RNA isolation.

### Arginase Activity Assay

Arginase activity in cell or tissue lysates was measured by the conversion of [^14^C-guanidino]-L-arginine to [^14^C]urea, which was converted to ^14^CO_2_ by urease and trapped as Na_2_^14^CO_3_ for scintillation counting [28], [29]. Arginase activity was expressed as nmoles urea formed per min per mg of protein at 37°C. The assay represents the sum of the activities when both arginase I and arginase II isozymes are present.

### Purification of Lung Endothelial Cells

Lung endothelial cells were isolated by an immunomagnetic isolation method, as previously described [30]. Only one round of selection was performed in which Dynabeads Sheep anti-Rat IgG (Invitrogen, catalogue number 110.35) coated with rat anti-mouse CD31 (PECAM-1, clone MEC13.3) antibody (Pharmingen Cat. No. 553369) were used, which resulted in EC rich and EC depleted fractions. After isolation adherent and non-adherent cells were homogenized in cell lysis solution [28] and the protein extracts obtained were used for analysis of arginase activity.

### Vascular Reactivity

Thoracic aorta and mesenteric arteries were excised and placed into ice-cold Kreb’s modified solution (composition in mM: NaCl 119, KCl 4.7, MgSO_4_•7H_2_0 1.17, NaHCO_3_ 25, KH_2_PO_4_ 1.18, CaCl_2_ 2.5, glucose 11 and EDTA 0.03). The adipose and connective tissue were removed and 2 mm length segments were mounted in the myograph. After an equilibration period of 30 min, all vessels were subjected to an oxygenated and pre-warmed (37°C) high potassium physiological salt solution (KPSS in mM; KCl 123, MgSO_4_•7H_2_0 1.17, NaHCO_3_ 25, KH_2_PO_4_ 1.18, CaCl_2_ 2.5, glucose 6.05 and EDTA 0.03) to assess vessel viability. Responses to vasodilators were then examined in arteries preconstricted to ∼50% KPSS with either cirazoline (10–100 nmol·L-1) or 30 mM potassium salt solution (30 mM KCl, composition in mM; NaCl 84, KCl 40, MgSO_4_•7H_2_0 1.17, NaHCO_3_ 25, KH_2_PO_4_ 1.18, CaCl_2_ 2.5, glucose 11 EDTA 0.03). Potassium was chosen for mesenteric arteries because at the concentration used, any effect of EDHF would be negated, unmasking the contribution of NO to vasodilatation [31]. All vasorelaxation responses were expressed as percent relaxation from the preconstriction response. Variable slope sigmoidal concentration response curves to each agonist were fitted and graphed, and the -log EC_50_ M value, (i.e. the concentration giving 50% of the maximum response) was calculated for individual curves using GraphPad Prism (v 5.0). R_max_ depicts the maximum relaxation response obtained.

### Measurement of Aortic NO Levels

NO was measured in aortas by measuring total nitrate and nitrite (NOx) levels using the Griess reaction method according to a kit (Cayman Chemical) as previously described [32]. Briefly, aortas were homogenized in phosphate buffered saline (PBS) pH 7.4 and the homogenate filtered using a 10 kDa Amicon filter. Aortic homogenates were incubated with nitrate reductase enzyme and enzyme cofactor mixture for 3 hours at room temperature. Griess reagent 1 and 2 were added to the homogenates and left to incubate at room temperature for 10 mins. Absorbance was read at 540 nm and the concentrations of NO were calculated from a standard curve and normalized to protein concentration.

### Measurement of Aortic Superoxide Level

Superoxide formation was measured using both L-012 enhanced chemiluminescence and dihydroethidium (DHE; 2 µM) staining [33], [34]. Thoracic aorta from 10–15 week old WT and hArgII transgenic mice were isolated and placed in ice-cold Krebs-HEPES solution and cleared of connective tissue. For the L-012 enhanced chemiluminescence, the aortae were segmented into lengths of 3–5 mm and incubated at 37°C in the dark for 1 hour. Background luminescence, read using a luminometer (Berthold, LB96), was subtracted from an average of 10 readings and normalized to dry weight. Each measurement was expressed as relative light units per second per mg of protein (RLU.sec^−1^.mg^−1^). For the DHE staining, tissues were immersed in Tissue-Tek as previously described [34] and snap frozen in liquid nitrogen and stored at −80°C until they were cryocut into 30 µM thick sections and mounted onto slides (Menzel-Glaser SuperFrost Plus). A Zeiss 510 Meta confocal microscope equipped with a krypton/argon laser was used to image the fluorescence of 2-hydroxyethidium, the specific product of the reaction. The laser settings were identical for each image acquired, excitation and emission spectra of 488 and 543 nm, respectively. The intensity of the fluorescence was quantified in three consecutive segments from the same aorta using Image J (version 1.42q) and the values averaged.

### Blood Pressure Determination

Mice were anesthetized with xylazine (6 mg/kg body wt i.p.) and ketamine chloride (120 mg/kg body wt i.p.) and placed in a supine position for measurement of blood pressures (systolic, diastolic and mean arterial) via a Millar-solid state pressure catheter (SPR-671, Millar Instruments Inc.) surgically inserted through the right carotid artery. Anesthesia and core body temperature (36.5–37 degrees Celsius) were maintained for the duration of the experiment. Only stable blood pressure data were included. Data was collected and analysed using Chart 5 Pro (ADI Instruments).

### Atherosclerotic Lesion Assessment

For evaluation of the development of atherosclerosis in experimental and control animals, we used *en face* measurements of the surface of mouse aortas covered by lipid deposits, as previously described [35] with aortas stained by Sudan IV. Quantification of plaques of each animal was performed in a blind fashion, using Image-Pro Plus version 4.1 software (Media Cybernetics, Inc., MD).

### Data Analysis and Statistics

Unless otherwise indicated, all data are presented as mean ± standard error of the mean (SEM). Results were analysed by Student *t-*test (paired or unpaired, as appropriate). Where three values were compared, comparisons were made using one-way ANOVA with Bonferroni posthoc analysis. Statistical analysis was performed using GraphPad Prism; *P*<0.05 was considered statistically significant.

## Results

### hArgII Mice

Two different founders of hArgII transgenic mice were generated: line 4, containing 120 copies of the transgene, and line 11 containing 60 copies in the heterozygous state, as determined by real-time qPCR genotyping. Relatively high levels of expression of hArgII transgene was found in all studied tissues examined compared to the endogenous mArgII gene (Table 1). In hArgII mice, the ratio hArgII to mArgII expression varied from almost 6-fold (in kidney) to more than 4800-fold and 6700-fold in aorta and heart, respectively. These observations were confirmed in Western blot analysis (Fig. 1A). Overexpression of hArgII in endothelial cells did not influence mArgI or mArgII expression when measurements were performed with RNA isolated from heart, kidney, liver and lung (Table 1). Both eNOS and iNOS mRNA expression were significantly increased in aorta from hArgII mice (P<0.05; n = 6; Fig. 1B&C) when compared to their non-transgenic littermate wild type (WT) controls, but there was no difference in protein expression (Fig. 1D&E; n = 4; P>0.05) nor the eNOS:monomer: dimer ratio (Fig. 1F; n = 6; P>0.05).

### Arginase Activity is Increased in hArgII Mice

**Figure 2:**
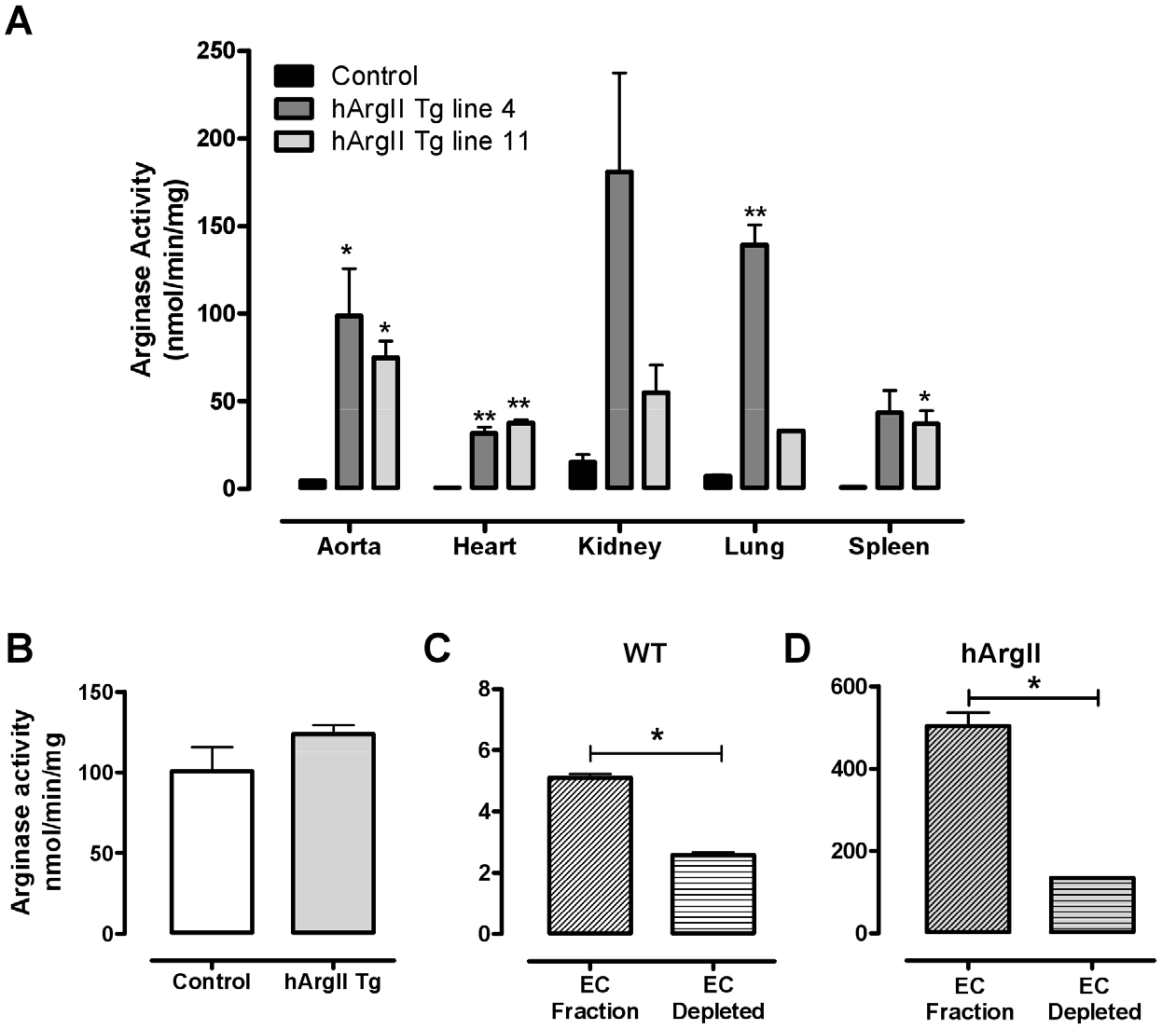
Arginase activities of: A heterozygous hArgII Line 4 and Line 11 mice and C57Bl/6 males (n = 3−5) and B resident peritoneal macrophages. Macrophage activities are mean and range for two groups each of pooled samples for male and female homozygous hArgII mice and littermate controls (n = 4/group for WT; n = 4 or 5/group for hArgII lines 11 and 4, respectively). Arginase activity in extracts from lung endothelial cells immunomagnetically separated to endothelial cells (EC fraction) or fractions depleted of endothelial cells (EC depleted) from **C** C57Bl/6 (n = 4) and **D** from hArgII Line 4 mice (n = 4). Data are presented as mean ± SEM. Values were compared **A** with a 1-way ANOVA with Bonferroni posthoc test and **B**–**D** with Student’s t-test where *  =  P<0.05, **  =  P<0.01 and ***  =  P<0.001.

The transgenic mice had elevated tissue total arginase activity when compared to their non-transgenic siblings: 5–40 fold in aorta, heart, kidney, lung and spleen (Fig. 2A). There was no difference in total arginase activity in the liver in hArgII mice (Line 4, 7793±568; Line 11, 9192±1027 vs. WT, 8510±667 nmol/mg/min; n = 3−5; P>0.05).

Specificity of the expression of the hArgII gene driven by the Tie2 promoter was verified by assessing the arginase activity of resident peritoneal macrophages and lung fractions either after enrichment or depletion of endothelial cells. Resident peritoneal macrophages from transgenic and control mice did not differ in the level of arginase activity (Fig. 2B). Furthermore, lung endothelial cell-enriched fractions isolated from hArgII transgenic animals had 3.7±0.3-fold higher arginase activity than fractions partially depleted of lung endothelial cells (P<0.05; Fig. 2C & D).

### Plasma L-arginine Metabolites and Lipid Levels are Unchanged

To assess the effect of the hArgII transgene, plasma concentrations of L-arginine and several of its metabolites were determined. Overexpression of hArgII did not lead to significant changes in plasma levels of L-arginine or its metabolites in transgenic mice compared to their non-transgenic littermate controls (data not shown). Furthermore, when the ratio of plasma arginine to the sum of plasma ornithine plus citrulline was determined, as an indicator of global arginine bioavailability, there were also no differences in the available plasma arginine bioavailability ratio (arginine/orthinine + citrulline) between strains (WT: 0.59±0.06, hArgII: 0.64±0.04; P>0.05; n = 6−11). Similarly, plasma lipid profiles were unaffected in the hArgII lines (data not shown).

### hArg II Overexpression Induces Endothelial Dysfunction

**Figure 3:**
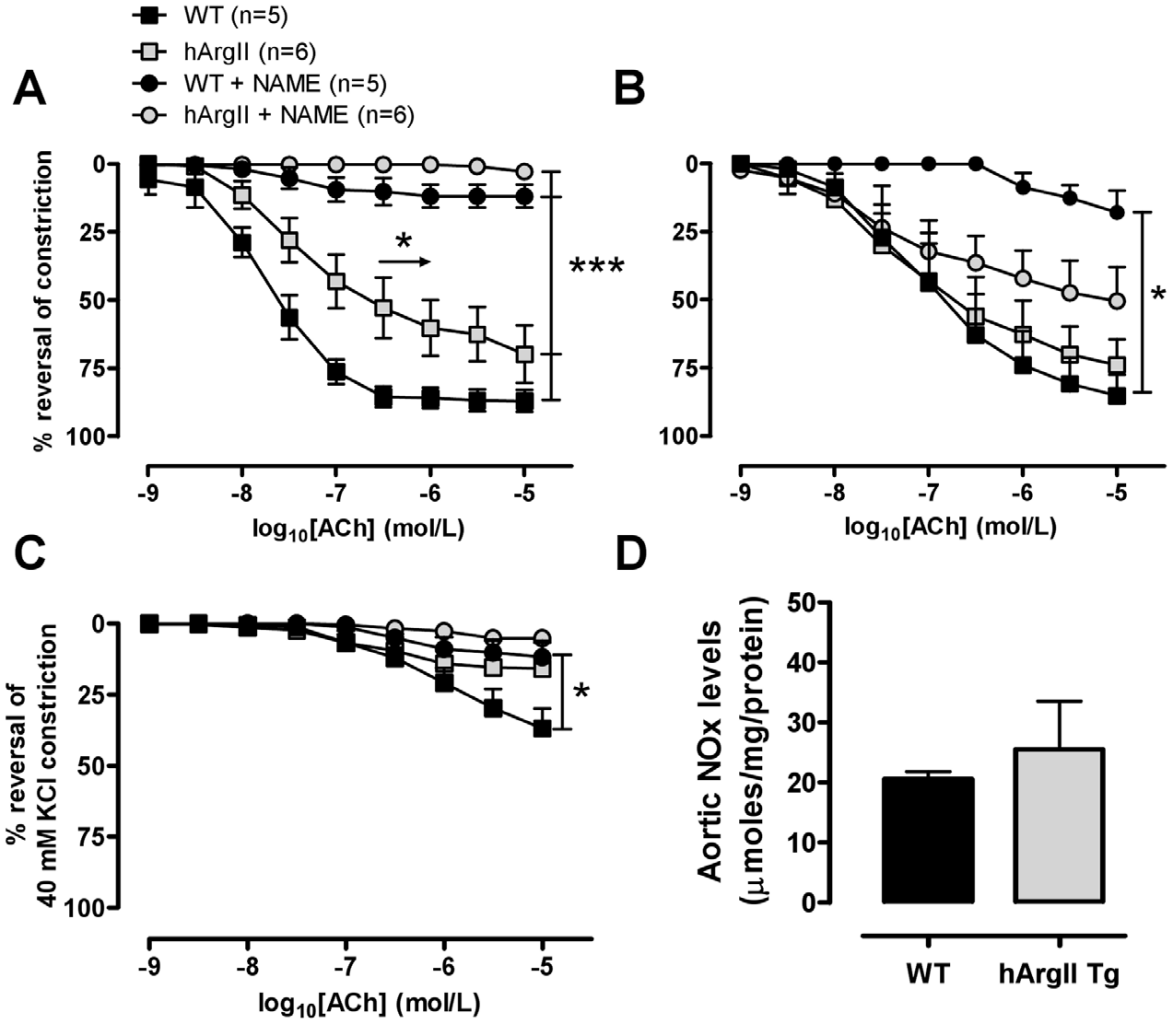
Concentration response curves to the endothelium-dependent vasodilator acetylcholine (ACh) before and after the addition of the nitric oxide synthase inhibitor NG-nitro-L-arginine methyl ester (NAME; 100 µmol/L) in A aorta constricted with cirazoline and mesenteric arteries constricted with B cirazoline or C high potassium from male homozygous hArgII Line 11 mice (n = 4−6) and their WT littermate controls (WT; n = 5−6). **D** NO production as determined by measuring NOx (nitrate and nitrate) using the Griess reaction in homogenized aorta and normalized to protein concentration in aorta from homozygous hArgII line 11 males and littermate non-transgenic control males (n = 8−9). Data are presented as mean ± SEM. EC_50_ and E_max_ values were compared using 1-way ANOVA with Bonferroni posthoc test, where * =  P<0.05 and *** =  P<0.001.

Maximal contractile responses to high K^+^ levels in aorta and small mesenteric arteries (SMA) from hArgII mice were not significantly different from their non-transgenic littermate controls (Table 2; n = 7−8; P>0.05). Endothelial dysfunction was evident in aortas from hArgII mice, as demonstrated by a significant shift to the right in the endothelium-dependent vasodilator acetylcholine (ACh)-induced relaxation (EC_50_: 7.1±0.2 M) compared to their non-transgenic littermate controls (EC_50_: 7.6±0.1 M; Fig. 3A, n = 5−8; P<0.05). There was no significant difference in ACh-mediated relaxation in small mesenteric arteries (SMA) constricted with cirazoline (Fig. 3B) or with inhibition of endothelium-dependent hyperpolarizing factor (EDHF) with a high K^+^ constriction (Fig. 3B; n = 4−7; P = 0.06), although the latter did tend towards statistical significance. The NOS inhibitor L-NAME (100 µM) attenuated the responses to ACh in aorta (Fig. 3A; n = 5−6; P<0.001) from WT and hArgII, confirming the NO dependency of the responses. L-NAME attenuated the responses to ACh in WT mice preconstricted with cirazoline and K^+^ (P<0.05), but there was no reduction in ACh responses from SMA in hArgII mice, suggesting an attenuated NO-dependent and increased EDHF-dependent response (Fig. 3B&C, n = 4−6; P>0.05).

**Table 2:** Responses to noradrenaline (NA) and sodium nitroprusside (SNP) in the absence and presence of the NOS inhibitor L-NAME (100 µM) in aorta and small mesenteric arteries from male homozygous Line 11 hArgII mice and their male WT littermate controls.

Agonist	KPSS	NA		NA + L-NAME		SNP		SNP + L-NAME		
Aorta	E_max_ (g)	EC_50_	E_max_ (g)	EC_50_	E_max_ (g)	EC_50_	R_max_	EC_50_	R_max_	n
hArgII Tg	0.8±0.1	8.1±0.1	0.49±0.1	8.5±0.1**	1.2±0.2*	8.1±0.2	87±7	8.6±0.2	98±1	6
WT	0.77±0.1	8.1±0.1	0.58±0.1	8.5±0.1**	1.3±0.1***	8.3±0.2	98±2	8.6±0.1	98±1	5–7
**SMA**										
hArgII Tg	0.50±0.1	6.6±0.4	1.46±0.3*	6.7±0.2	1.37±0.3	7.5±0.1	92±7	7.7±0.4	92±8	4–5
WT	0.9±0.2	6.3±0.1	0.68±0.1	6.3±0.1	0.68±0.1	7.9±0.1	98±2	7.8±0.4	98±1	4–5

EC_5O_ values are expressed as –log M, E_max_ values as the maximum response to NA in g, R_max_ values as % reversal of the level of pre-contraction in response to 10 µM of the SNP. Values are given as mean ± SEM. n = 4−8 per group.

* *P<*0.05,

** *P<*0.01,

*** *P<*0.001 vs WT control (Student’s t-test).

In the aorta, no differences were observed between the different strains in responses to the endothelium-independent vasodilator sodium nitroprusside or the vasoconstrictor noradrenaline (NA) (Table 2; n = 5−8; P>0.05). Denudation of the endothelium (data not shown) or treatment with L-NAME enhanced the constriction to NA, although the magnitude of augmentation was similar between all strains of mice (Table 2; n = 5−8; P>0.05). Furthermore, there was significantly enhanced vasoconstriction in SMA from hArgII mice compared to WT, which was not affected by NOS inhibition (Table 2; n = 4−5; P<0.05).

### Basal Aortic NO Levels are Unaffected by hArgII Overexpression

NO production assessed by the Griess reaction showed no differences in the production of NO in aorta of hArgII when compared to their WT littermate controls (n = 8−9; P<0.01; Fig. 3C).

### ROS Levels are Unchanged in hArgII Aorta

ROS levels, assessed by both L-012-enhanced chemiluminescence and dihydroethidium (DHE) staining were unaltered in the aortas of hArgII (DHE: 3299±11; L-012: 192±43) mice versus their WT littermate controls (DHE: 3390±68; L-012: 180±40 relative light units; n = 4−6; P>0.05).

### Blood Pressure is Increased in hArgII Mice

**Figure 4:**
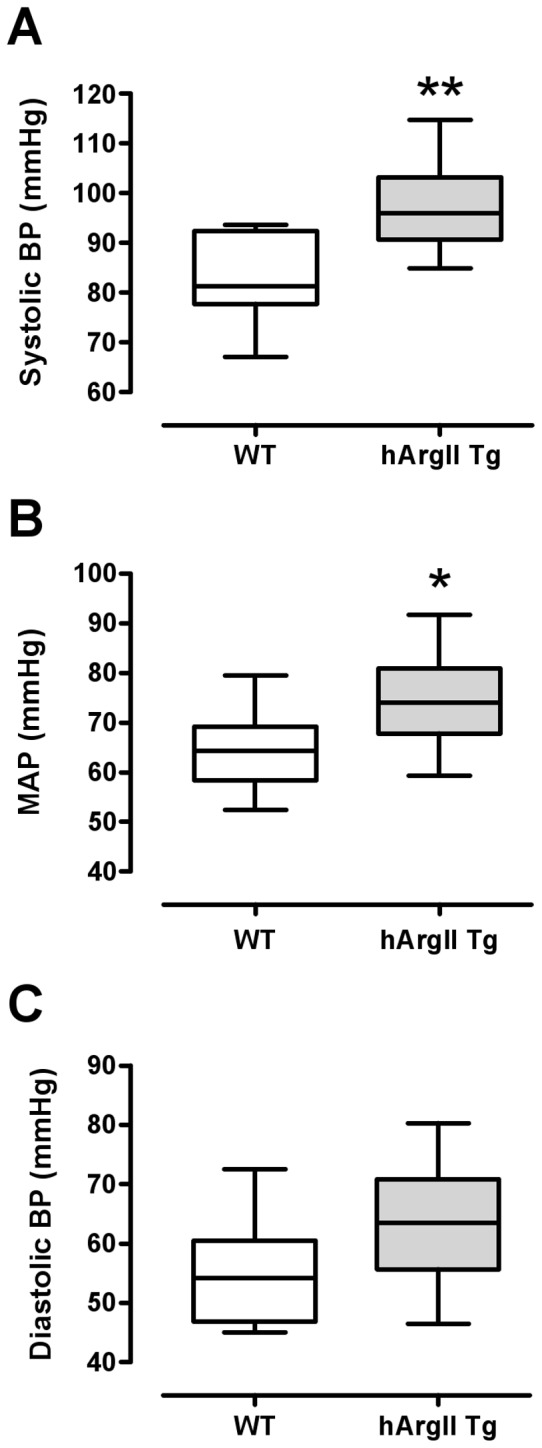
Blood pressure of anaesthetized hArgII and non-transgenic mice presented as box plots of A systolic blood pressure (systolic BP), B mean arterial pressure (MAP) and C diastolic BP. The box extends 25^th^ percentile to the 75^th^ percentile with the median represented by the line within the box. Measurements were from control non-transgenic sibling males (n = 9) versus Line 4 and 11 hArgII males (n = 9). Values were compared using unpaired Student’s t-tests where *  =  P<0.05 and **P * = * <0.01.

Arterial blood pressure, monitored by a Millar-solid state pressure catheter surgically inserted into the right carotid artery, indicated that hArgII mice had 17–20% increases in systolic and mean arterial blood pressure when compared to their non-transgenic littermate controls (n = 10; P<0.05; Fig. 4A, B). Although not statistically significant, diastolic blood pressure tended to be increased (P = 0.06; Fig. 4C).

### Atherosclerotic Lesions in hArg/apoE −/− Cross are Enhanced

**Figure 5:**
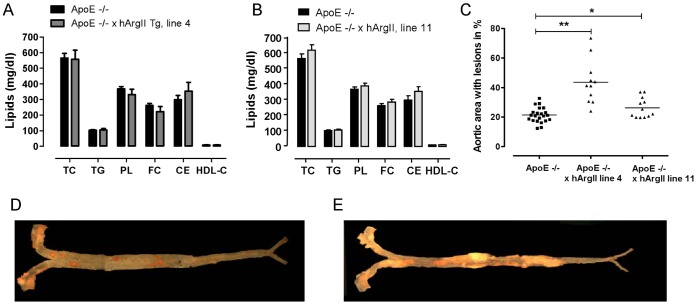
Plasma lipid profiles including total cholesterol (TC), triglycerides (TG), phospholipids (PL), free cholesterol (FC), cholesteryl esters (CE) and high density lipoprotein cholesterol (HDL-C) for female 8-month-old A ApoE −/− sibling control (n = 21) and heterozygous and homozygous Line 4 hArgII x ApoE −/− mice (n = 9) or B ApoE −/− sibling control (n = 21) and heterozygous and homozygous Line 11 hArgII x ApoE −/− mice (n = 19). C Plaque lesion area in aorta from 8-month-old female ApoE −/−sibling control (n = 22) and heterozygous Line 4 hArgII x ApoE −/− females (n = 11) and heterozygous and homozygous Line 11 hArgII x ApoE −/− females (n = 12). Images of en face Sudan IV staining of atherosclerotic plaque in the whole aorta from 8-month-old **D** heterozygous Line 4 female hArgII x ApoE −/− and E ApoE −/− sibling control mouse. Data are presented as mean ± SEM. Values were compared with either a unpaired Student’s t-test or a 1-way ANOVA with Bonferroni posthoc test where *  =  P<0.05 and **  =  P<0.01.

In order to examine the effect of the hArgII transgene on atherogenesis, the transgenes were transferred to mice with an apoE −/− background. Insertion of the hArgII transgene did not affect total cholesterol, triglycerides or HDL in 8-month-old crossed hArgII/apoE −/− mice when compared to comparably aged control apoE −/− mice (Fig. 5A, B; n = 8−13; P>0.05). Nevertheless, e*n face* aortic lesion measurement throughout the aorta with Sudan IV lipid staining demonstrated that 8-month-old hArgII/apoE −/− females had significantly increased aortic lesion development in both lines 4 and 11 compared to apoE−/− sibling control females (Fig. 5C; n = 11−22; P<0.05). The effect of the transgene was dependent on the level of arginase activity in tissues. Transgenic mice belonging to line 4, the strain exhibiting the highest arginase activity in tissues (see Fig. 2A), had twice the development of aortic lesions in comparison with control (P<0.001). Some transgenic mice had greater than 70% surface area coverage by atherosclerotic plaque (Fig. 5D), whereas apoE −/− mice typically only had 20% surface area coverage (Fig. 5E).

## Discussion

In the current study, we show that the overexpression of the arginase II isoform elicits hypertension and exacerbates atherogenesis. Our results suggest that overexpression of hArg II leads to endothelial dysfunction via a decrease in NO production and that this contributed to hypertension and increased lesion development in the transgenic mice.

Consistent with previous reports, [36] the Tie2 promoter drove overexpression of the hArgII transgene in endothelial cells, as indicated by the marked enrichment of arginase activity in isolated lung endothelial cells (Fig. 2D). Likely dependent on the site of integration into chromosomal DNA, the Tie2 promoter has, at least in one case, been found to drive expression of a transgene in myeloid cells [37]. Expression of the hArgII transgene was therefore evaluated in peritoneal macrophages. In marked contrast to endothelial cells, there was no overexpression of hArgII in macrophages (Fig. 2B). The hArgII transgene did not significantly alter the expression of the mouse endogenous genes for arginase I and II in any of the tissues analysed (Table 1), rendering it unlikely that the observed vascular effects resulted from changes to endogenous arginase.

There were no significant differences between WT and the transgenic mice in the plasma levels of L-arginine or its metabolites, indicating that elevated endothelial arginase activities did not have a significant impact on the circulating arginine pool. The lack of differences in plasma L-arginine levels does not, however, necessarily indicate that there were no decreases in endothelial L-arginine levels in the hArgII arginase transgenic mice. The production of NO from its substrate L-arginine is also known to be dependent on the distribution and stimulation of eNOS. Functional changes in eNOS activity and/or enhanced oxidative stress, rather than reduced eNOS expression, may be responsible for NO inactivation in atherosclerosis. Indeed, although somewhat counterintuitive, in apoE −/− mice, eNOS mRNA expression is either unchanged or even increased, [38], [39] as was observed in the hArgII mice in the present study (Fig. 1B). This has been reported elsewhere in the literature, where increases in arginase II [11] in endothelial cells are associated with decreased NOS activity and NO production in the presence of increased or unchanged expression of eNOS and iNOS, as demonstrated in the current study. Furthermore, since the uncoupling of eNOS from its dimeric state has been associated with increased arginase expression and ROS production, [40] the monomer:dimer ratio of eNOS was determined. Although somewhat unexpected, there was no significant difference in the ratio between the hArgII transgenics and their WT controls.

To determine the effect of endothelial overexpression of arginase II on NO bioavailability, responses to the endothelium-dependent vasodilator ACh were examined in aortae and small mesenteric arteries. Overexpression of hArgII significantly reduced ACh-induced NO responses in aortae and inhibition of NOS with L-NAME confirmed the NO dependence of the response, suggesting the regulation of NO production via arginase II. There were no differences observed in mesenteric arteries preconstricted with cirazoline. However, the response to ACh in mesenteric arteries preconstricted with cirazoline, can be mediated by both NO and EDHF. In WT mesenteric arteries constricted with cirazoline, NOS inhibition markedly attenuated the response, indicating that the relaxation was predominantly NO-mediated. The lack of effect of NOS inhibition on responses in mesenteric arteries from the hArgII mice (Fig. 3B) suggests that the response was mediated predominantly via EDHF. Indeed, when a high potassium solution was used to negate the EDHF response induced by ACh, the response to NO in the hArgII mice was negligible compared to the WT NO response (Fig. 3C) that were ‘unmasked’ by EDHF inhibition. These findings are in agreement with others in the literature, [41], [42] indicating that arginase II can regulate NO bioavailability in endothelial cells and demonstrates that increases in arginase II reduce endothelial function.

ROS production was measured in aortae of the transgenic mice, and no significant difference in basal ROS production was observed. Although increases in ROS have been reported in atherosclerosis, wherein there is increased vascular arginase activity, [11] some of which may be induced by NADPH oxidase-generated ROS, [43] or uncoupled eNOS [44] the results from the present study are aligned with the unchanged eNOS monomer:dimer ratio findings and do not suggest a converse role for arginase in modulating ROS production.

Decreased NO production often accompanies high blood pressure [45], [46] and increased arginase expression and activity have been well-documented in this disease state. [5], [47] Consistent with these reports, our findings demonstrate a critical role for arginase II in the development of hypertension.

NO protects endothelial function by reducing inflammation, smooth muscle proliferation, leukocyte adhesion and platelet aggregation. Endothelial dysfunction, due to decreased NO, has been extensively reported in humans and animal models of atherosclerosis [48]. Taken together with reports of increased arginase activity and expression in atherosclerosis and the decreased NO bioavailability of the hArgII transgenic mice we explored the role of arginase in the development of atherosclerosis in crossed hArgII transgenic mice with apoE −/− mice. Overexpression of hArgII in endothelium significantly increased aortic atherosclerotic lesion area in these mice. Lesion development was greatest in the line 4 hArgII transgenics. Plasma lipids from the crossed ApoE−/− and hArg transgenics were similar to their non-transgenic apoE −/− littermates, indicating a lipid-independent effect on lesion development. Whilst ROS were unchanged and NO bioavailability was reduced in the hArgII transgenic mice the effect of increased hArgII transgene expression in mice on an apoE −/− background warrants further investigation. However, when noted in the context of the effects elicited by the hArgII enzyme on endothelial function, the apparent lipid-independent atherosclerotic lesion development may indicate a role for endothelial eNOS interaction with hArgII expression.

In summary, mice with endothelial-specific overexpression of human arginase II were generated and characterized with respect to vascular pathophysiology. Increased arginase II expression appears to result in decreased NO bioavailability, which augments MAP. Furthermore, when the hArgII transgenic mice were crossed with apoE −/− mice, we observed increased atherosclerotic plaque development. Importantly, increasing endothelial arginase II expression, in the absence of any changes in plasma lipid levels, is sufficient to increase plaque development, providing strong evidence for the critical role of arginase II in the development and progression of atherosclerosis.
